# A mixed methods study to assess the feasibility of a randomised controlled trial of invasive urodynamic testing versus clinical assessment and non-invasive tests prior to surgery for stress urinary incontinence in women: the INVESTIGATE-I study

**DOI:** 10.1186/s13063-015-0928-2

**Published:** 2015-09-08

**Authors:** Paul Hilton, Natalie Armstrong, Catherine Brennand, Denise Howel, Jing Shen, Andrew Bryant, Douglas G. Tincello, Malcolm G. Lucas, Brian S. Buckley, Christopher R. Chapple, Tara Homer, Luke Vale, Elaine McColl, on behalf of the INVESTIGATE studies group

**Affiliations:** Newcastle upon Tyne Hospitals NHS Foundation Trust, Newcastle upon Tyne, UK; Department of Health Sciences, University of Leicester, Leicester, UK; Newcastle Clinical Trials Unit, Newcastle University, Newcastle upon Tyne, UK; Institute of Health & Society, Newcastle University, Newcastle upon Tyne, UK; Department of Urology, Morriston Hospital, Swansea, UK; School of Medicine, National University of Ireland, Galway, Ireland; Department of Urology, The Royal Hallamshire Hospital, Sheffield, UK; Royal Victoria Infirmary, Level 5, Leazes Wing, Newcastle upon Tyne, NE1 4LP UK

**Keywords:** feasibility studies, pilot studies, interview studies, randomised controlled trial, stress urinary incontinence, urodynamics

## Abstract

**Background:**

The position of invasive urodynamic testing (IUT) in diagnostic pathways for urinary incontinence is unclear, and systematic reviews have called for further trials evaluating clinical utility. The objective of this study was to inform the decision whether to proceed to a definitive randomised trial of IUT compared to clinical assessment with non-invasive tests, prior to surgery in women with stress urinary incontinence (SUI) or stress-predominant mixed urinary incontinence (MUI).

**Methods:**

A mixed methods study comprising a pragmatic multicentre randomised pilot trial, a qualitative face-to face interview study with patients eligible for the trial, an exploratory economic evaluation including value of information study, a survey of clinicians’ views about IUT, and qualitative telephone interviews with purposively sampled survey respondents. Only the first and second of these elements are reported here.

Trial participants were randomised to either clinical assessment with non-invasive tests (control arm) or clinical assessment with non-invasive tests plus IUT (intervention arm).

The main outcome measures of these feasibility studies were confirmation that units can identify and recruit eligible women, acceptability of investigation strategies and data collection tools, and acquisition of outcome data to determine the sample size for a definitive trial. The primary outcome proposed for a definitive trial was ICIQ-FLUTS (total score) 6 months after surgery or the start of nonsurgical treatment.

**Results:**

Of 284 eligible women, 222 (78 %) were recruited, 165/219 (75 %) returned questionnaires at baseline, and 125/200 returned them (63 %) at follow-up. Most women underwent surgery; management plans were changed in 19 (19 %) participants following IUT.

Participants interviewed were positive about the trial and the associated documentation.

**Conclusions:**

All elements of a definitive trial were rehearsed. Such a trial would require between 232 and 922 participants, depending on the target difference in the primary outcome. We identified possible modifications to our protocol for application in a definitive trial including clarity over inclusion/exclusions, screening processes, reduction in secondary outcomes, and modification to patient questionnaire booklets and bladder diaries. A definitive trial of IUT *versus* clinical assessment prior to surgery for SUI or stress predominant MUI is feasible and remains relevant.

**Trial registration:**

Current Controlled Trials: ISRCTN 71327395, registered 7 June 2010.

## Background

Urinary incontinence (UI), whilst rarely life-threatening, may seriously influence the physical, psychological and social wellbeing of affected individuals [1–4]. The impact on families and carers may be profound and the resource implications for health services considerable [5]. Prevalence figures for UI range from 5 % to 69 % in women 15 years and older, with most studies showing prevalence in the range 25 to 45 % [6]; stress (SUI) or mixed urinary incontinence (MUI) account for 65–85 % of cases [7].

Several methods are used in the assessment of UI to guide management decisions; some of these are non-invasive (for example, urine culture, bladder diaries or frequency volume charts, urine flow rate and post-void residual volume measurement), and some are invasive (that is, require catheterisation). Cystometry, the most commonly used invasive urodynamic test (IUT), looks at the pressure/volume relations during bladder filling, storage and emptying, with a view to defining a functional diagnosis as distinct from a purely symptomatic one.

The current position of IUT in the diagnostic pathway is not agreed upon, and practices vary considerably; in a UK survey in 2002, only half of the units surveyed had a guideline on indications for the tests, and 85 % carried out cystometry in all women with incontinence [8]. Current guidance from the National Institute for Health and Care Excellence (NICE), however, suggests that cystometry is not required prior to conservative treatments for UI, nor prior to surgery where the diagnosis of SUI is clear on clinical grounds (that is, where there are no symptoms of overactive bladder (OAB) or voiding dysfunction, no anterior compartment prolapse, and no previous surgery for SUI) [9–12].

Changes in available operative techniques, and in particular the introduction of less invasive approaches such as mid-urethral tapes, have resulted in dramatic alterations to surgical practice in recent years [13]. Hospital Episode Statistics (HES) demonstrated a 50 % increase in surgery for SUI in the 10 years following the introduction of mid-urethral tapes in 1997, with numbers apparently plateauing at 11,000 to 13,000 procedures annually in England between 2006 and 2007 and between 2012 and 2013 [14]. Were the NICE guidance to be applied, the annual savings from more rational use of IUT prior to surgery for SUI, based on 2012/13 national tariff costs (£403 per procedure for Healthcare Resource Group LB42Z) [15] and HES activity data [14], would be approximately £3.3 million. There would also be an additional ‘opportunity cost’ savings from the alternative use of staff and equipment currently devoted to IUT. On the other hand, it must be recognised that there are increasing concerns about the long-term safety of vaginal mesh implants [16], which might argue more in favour of increasing use of investigation to ensure the most rational use of surgery.

Two trials looking at the clinical utility of urodynamics in women with SUI have been published recently, both using a non-inferiority design. The VUSIS-1 trial from the Netherlands was terminated prematurely due to slow recruitment after achieving only 23 % (59/260) of its planned accrual [17]. In view of the recruitment difficulties with VUSIS-1, the group proceeded to a further study of alternative design, (VUSIS-2) in which all women underwent IUT, and only those with discordant clinical and urodynamic findings were randomised between surgical treatment (as dictated by their clinical assessment) and individualized treatment (dictated by the combination of clinical and urodynamic results); neither participants nor healthcare professionals involved were blinded to the urodynamic results in either group [18].

The ValUE trial from the USA defined a non-inferiority margin of 11 % [19]; this is equivalent to a standardised difference of <0.8, which may be considered high in statistical terms. [20] A difference in outcome between groups of 11 % may also be considered important in clinical terms, potentially influencing the decisions of both clinicians and patients. Notwithstanding these limitations, both studies reported that, in women with uncomplicated SUI, treatment (usually an immediate mid-urethral sling operation) based on basic clinical evaluation is not inferior to individually tailored treatment based on urodynamic findings.

Each of these studies was published during the period of recruitment and follow-up in INVESTIGATE-I [17, 19, 21]. How much they have already influenced clinical opinion and practice, or will do so in the future, is unclear, although a ‘point-counterpoint’ debate published after these studies makes it clear that there is still a question to be answered [22, 23]. The most recent update of the Cochrane review of urodynamics for the management of urinary incontinence in children and adults included the data from these two trials, yet continued to emphasise the need for larger definitive trials, in which people are randomly allocated to management according to urodynamic findings or to standard management based on history and clinical examination [24]. In addition to NICE [9–12] and the Cochrane Collaboration [24], the National Institute for Health Research - Health Technology Assessment programme (NIHR-HTA) [25] and the International Consultations on Incontinence (ICI) [26, 27] have also reviewed research literature on urodynamics, and, along with the James Lind Alliance Urinary Incontinence Priority Setting Partnership [28, 29], have called for high quality primary research assessing their clinical utility.

But several considerations indicated the need for a pilot trial and feasibility assessment before undertaking a definitive trial. The first consideration is the calculation of an informed sample size. Calculations based on estimates and assumptions from previously published modelling exercises [9, 30] and a previous surgical trial [31, 32] are sensitive to parameter values such as the proportion of recruits with SUI [30], the proportions of poor outcomes in the two arms, and the effect size (target difference) of interest. Calculations based on data in the most recent Cochrane review of urodynamics indicates that a sample size of over 1,600 per arm would be required to address this question [24]. Therefore, given the possible size and cost of a definitive trial, a pilot trial was considered crucial to test the assumptions made, give relevant estimates of key parameters, and ensure that a definitive trial would represent value for money from public funds. Secondly, a feasibility assessment could establish whether sufficient clinicians are willing to randomise patients within a definitive trial. IUTs have been widely used in clinical practice over the last 30 years, and despite the lack of evidence of clinical utility, many clinicians look on cystometry as a mandatory part of the investigation of patients with UI, particularly prior to surgical treatment [33–35]. A survey of members of the British Society of Urogynaecology (BSUG) has shown a high level of disagreement with the NICE guidance in this respect [36], and others have questioned the safety of the recommendations [37]. Finally, a key feasibility objective was to assess patient willingness to participate and identify barriers to and facilitators of participation. Patients may not so easily see the importance of ‘testing a test’ in the same way as they might view testing a treatment. Women may be willing to undergo even invasive investigation [38] in the belief that this will inevitably guide them and their clinicians towards appropriate treatment, and away from inappropriate and possibly harmful interventions. In a pilot patient preference study, only 32 % of the women were prepared to be randomised [38].

Recognising that a pilot randomised controlled trial (RCT) alone was probably inadequate to address the complexities of feasibility for a definitive trial in this aspect of healthcare, the INVESTIGATE-I study comprised an external pilot RCT, an exploratory health economic analysis and value of information study, a national survey of relevant clinicians, and separate qualitative interview studies with patients eligible for the trial and clinicians responding to the survey. Only the first and second of these elements are reported here.

The original study protocol was published in this journal [39]; two later amendments were approved by the Research Ethics Committee, and the final version of the protocol (v1.2) is available on the NIHR website http://www.nets.nihr.ac.uk/projects/hta/0922136. The clinician survey and interview study have been published in full previously [40, 41], and a separate publication is planned for the economic evaluation and value of information study [42]. This report therefore, whilst drawing conclusions from the whole collection of studies, focuses on the pilot trial itself, and the qualitative interview study with trial participants.

## Methods

The conduct of this study was in accordance with the ethical principles set out in the Declaration of Helsinki (2008) and the Research Governance Framework for Health and Social Care (second edition, 2005) [43]. Application for ethical approval was made through the Integrated Research Application System (IRAS), and a letter of favourable ethical opinion was obtained from Newcastle & North Tyneside 1 Research Ethics Committee on 6th January 2011 - reference no. 10/H0906/76. All elements of the study were approved by local Research and Development offices at Newcastle upon Tyne Hospitals NHS Foundation Trust (28/03/2011), Gateshead Health NHS Foundation Trust (29/03/2011), Abertawe Bro Morgannwg University Health Board (23/06/2011), Sheffield Teaching Hospitals NHS Foundation Trust (07/07/2011), Northumbria Healthcare NHS Foundation Trust (25/07/2011), University Hospitals of Leicester NHS Trust (09/08/2011), City Hospitals Sunderland NHS Foundation Trust (30/05/2012), South Tees Hospitals NHS Foundation Trust (09/07/2012) and South Tyneside NHS Foundation Trust (17/09/2012); hence the favourable ethical opinion was applicable to all NHS sites taking part in the study.

The objective of the feasibility study (INVESTIGATE-I) was to inform the decision as to whether to proceed to a definitive RCT of the clinical and cost-effectiveness of IUT compared to basic clinical assessment with non-invasive testing in women potentially suitable for surgical treatment of SUI or stress predominant MUI and whether any refinements to the proposed definitive trial design were warranted [44–48].

### Pragmatic multicentre randomised pilot trial

The pilot RCT was designed to rehearse the methods and processes of any future definitive RCT.

#### Units recruiting to the trial

Recruitment to the pilot trial was initially limited to six specified units; these were a mix of specialist urogynaecology (Newcastle upon Tyne and Leicester) and female urology (Sheffield and Swansea) departments in university teaching hospitals, providing secondary and tertiary level care, and general gynaecology units in district general hospitals, providing secondary care services (Wansbeck Hospital, Northumberland, and Queen Elizabeth Hospital, Gateshead).

In order to improve adherence with recruitment targets and to test the processes for possible future use, two Patient Identification Centre (PIC) sites (Sunderland Royal Hospital and South Tyneside District General Hospital) and one additional full recruiting site (South Tees Hospitals NHS Foundation Trust) were added in 2012.

#### Inclusion and exclusion criteria

##### Inclusion criteria

Inclusion criteria for the pilot RCT (and anticipated inclusion criteria for any future definitive RCT) were as follows:

1. Clinical diagnosis of SUI or stress-predominant MUI.
2. Women must state that their family is complete.
3. Women should have undergone a course of pelvic floor muscle training (± other non-surgical treatments for their urge symptoms) with inadequate resolution of their symptoms.
4. Both the woman and her treating clinician should agree that surgery is an appropriate and acceptable next line of treatment.

##### Exclusion criteria

Exclusion criteria for the pilot RCT (and anticipated exclusion criteria for any future definitive RCT) were as follows:

1. Symptomatic utero-vaginal prolapse requiring treatment.
2. Previous surgery for urinary incontinence or pelvic organ prolapse.
3. Urodynamic investigation within the last three years.
4. Neurological disease causing urinary incontinence.
5. Current involvement in competing research studies, for example, studies of investigation or treatment of urinary incontinence.
6. Unable or unwilling to give competent informed consent.

#### Recruitment

Potential trial recruits were identified by research nurses prior to attending new or follow-up appointments for SUI or MUI. A short Patient Information Leaflet (PIL) was Xincluded with a letter of invitation, with new appointments or reminder letters for follow-up appointments. A full (6-page) PIL was provided on request. The study information was discussed at the first hospital visit; women declining to take part underwent further investigation and or treatment as clinically appropriate at the same visit. Written consent was obtained from those agreeing to take part, before randomisation. To ensure concealment of allocation, randomisation was undertaken by an internet-accessed computer randomisation system held by the Newcastle Clinical Trials Unit (NCTU); randomisation between intervention and control was 1:1, and was stratified by centre using random block length. It was neither feasible nor appropriate to blind participants or clinicians (investigating and operating) to the allocation of the investigation strategy.

#### Sample size

The sample size for the external pilot trial was determined pragmatically, using the recommended minimum of 30 participants per arm. [47] It was hoped that 60 would be retained per trial arm to investigate the distribution and key parameters of the outcome measures. Previous trials in the area of pelvic floor dysfunction, including investigation [49], surgical [32, 50, 51], and non-surgical treatments [52] suggested average attrition rates of 13 % (7–20 %) between identification and randomisation, 16 % (6–20 %) between randomisation and treatment, and 13 % (9–20 %) between treatment and follow-up at 6 months. Based upon the more pessimistic figure in each case, it was estimated that a total of 240 eligible patients should be approached, allowing for a 50 % overall attrition.

#### Interventions

Patients were randomised to receive either of the following:

1. No IUT - basic clinical assessment supplemented by non-invasive tests as directed by the clinician; these included frequency/volume charting or bladder diary, mid-stream urine culture, urine flow rate and residual urine volume measurement (by ultrasound), or
2. IUT - basic clinical and non-invasive tests as above, plus invasive urodynamic testing (IUT). Dual-channel subtracted cystometry with simultaneous pressure/flow voiding studies is the most commonly applied technique in the evaluation of patients prior to surgery for SUI in most centres; video-urodynamics and ambulatory bladder pressure monitoring were also permissible at the discretion of the clinician.

Further investigation was undertaken where appropriate at the same visit or a later one, as per local practice, and the treatment plan formulated.

#### Outcome measures

The collection of the outcome measures for a future definitive RCT was piloted, to assess data yield (for example, percentage of recruited participants returning completed questionnaires) and quality (for example, completeness and consistency of responses within returned questionnaires). This information was collected to guide the choice and mode of administration of questionnaires and data collection tools in any future definitive RCT.

The primary outcome rehearsed in the pilot RCT was a patient-reported outcome measure (PROM):

1. The combined symptom score of the International Consultation on Incontinence - female lower urinary tract symptoms questionnaire (ICIQ-FLUTS) at 6 months after treatment [31].

Secondary outcomes rehearsed were as follows:

1. General health questionnaire (SF-12v2™ Health Survey © 1994, 2002 by QualityMetric Incorporated and Medical Outcomes Trust) [53], and EQ-5D-3 L © 1990 by EurQol Group [54])
2. Quantification of urinary leakage (three day bladder diary, and ICIQ-UI SF) [55]
3. Prevalence of symptomatic *‘de novo*’ functional abnormalities including voiding dysfunction and detrusor overactivity (using subscales in ICIQ-FLUTS [31], with cystometric investigation in symptomatic patients)
4. The impact of urinary symptoms on quality of life (ICIQ-LUTSqol and UDI) [56, 57]; the latter measure was included since it was used in the VUSIS and VALUE trials [18, 19].
5. Use of health services and costs to the NHS and to patients

#### Baseline assessment of study outcomes

Following consent and randomisation, patients were given a pack of baseline study outcome questionnaires*.* Participants were asked to complete the questionnaires at home within 2 weeks of receipt and post them to the central trial office using a prepaid envelope.

#### Subsequent treatment within the trial

Following investigation, it was expected that women randomised to the ‘no IUT’ arm of the study would undergo surgical treatment. The choice of operation was left to the individual surgeon and woman; because only primary cases were included, it was anticipated that in most cases this would be either a retropubic or transobturator foramen mid-urethral tape procedure. It was expected that those randomised to the intervention ‘IUT’ arm would have similar surgical treatment when urodynamic stress incontinence (USI) was confirmed. Where other diagnoses were identified following investigation, alternative treatments might be offered, which were informed by which other conservative treatments had previously been tried. These included bladder retraining, anti-muscarinic drug treatments, neuromodulation, botulinum toxin injections (where detrusor overactivity (DO) was diagnosed), or clean intermittent self-catheterisation (where a voiding dysfunction was identified). In all centres, the treatment algorithm employed was in keeping with the then current NICE recommendations (2006) [9].

#### Follow-up

Clinicians arranged post-operative follow-up or other outpatient review, as per their normal practice and timing. Women were sent a pack of follow-up study outcome questionnaires and bladder diaries along with a prepaid envelope at 6 months after surgery, at the start of any non-surgical intervention, or at a period of ‘watchful waiting’. They were asked to complete and then post them to the central trial office. Those failing to return questionnaires within 1 month were contacted by a research nurse by telephone to encourage responses. In the last 9 months of the study, the option of completing the questionnaire over the telephone with the research nurse was also given to participants during the reminder telephone call. Those who did not return the questionnaires after a telephone reminder were sent a second copy of the questionnaires. Each patient’s withdrawal or completion of the study follow-up was documented in the case report form (CRF).

### Qualitative interviews with women eligible for the pilot trial

Interviews were carried out to explore the women’s understandings and experiences of the study, including the consent processes and their decision to participate. Purposive sampling was used to invite women from a range of ages, trial participation status (randomised and retained to final follow-up; randomised but did not provide full follow-up data), allocation status (IUT or basic assessment), treatment received (surgery or conservative management), and study site. It was also intended that women who declined randomisation would be interviewed.

Women were approached at the end of the trial so as to capture both their reasons for agreeing to participate and their overall experience of taking part in the study. A specific Participant Information Leaflet was provided for the interview study, and written consent was obtained from all interviewees. The interviews were carried out face-to-face by an expert qualitative interviewer (see acknowledgements) and were audio-recorded and transcribed verbatim.

The interviews were semi-structured, using a prompt guide with broad topic areas, but the emphasis was on encouraging women to discuss their own perspectives freely, thereby allowing them to raise issues that were important to them. The interviewer prompted as appropriate to ensure that all views were fully explained and the meaning of participants’ responses clear. The prompt guide was developed from a literature review and discussions within the project team and was modified as the interviews progressed to incorporate issues raised by earlier interviewees.

Analysis took place alongside data collection, which continued until saturation of themes was reached and interviews no longer generated new concepts. All completed interviews were included in the analysis. Analysis was based on the constant comparative method [58], and aided by *NVivo 10* software (© QSR International, Warrington, UK). Data analysis was carried out by an experienced qualitative researcher (see acknowledgements) under the supervision of NA. To maximise the credibility and rigour of the analysis, NA regularly reviewed the coding scheme and interview transcripts, and any differences in interpretation were discussed and reconciled. Further details of the methods are published in full in the protocol document [39, 59].

### Synthesis of findings

The analytic framework proposed by Bugge et al. [45] was used to summarize findings from the pilot trial and participant interviews; this framework comprises 14 methodological issues, derived from the work of Shanyinde et al. [60] on what needs to be evaluated in pilot and feasibility studies.

This analysis is followed by the 3-step ADePT process, involving:

1. Deciding on the type of problem experienced (Type A - the issue is likely to be a problem only for the trial; Type B - the issue is likely to be a problem for both the trial and the real world; Type C - the issue is likely to be a problem only for the real world), and the associated evidence;
2. Identifying the range of possible solutions and the evidence to support those solutions, including assessment of the potential effectiveness and potential feasibility of each option;
3. Assessing the best options.

## Results

The summary of methodological issues [60], and their analysis after Bugge et al. [45], is given in Table 1.

**Table 1:** Summary of findings against 14 methodological issues for feasibility research

Methodological issue	Findings	Evidence
1. Did the feasibility/pilot study allow a sample size calculation for the main trial?	Achieved: a definitive trial would require recruitment of between 232 (for a target difference of four units in ICIQ-FLUTS score at 6 months) and 922 (for a target difference of two units) patients, to achieve a sample of 130 to 516 primary outcome responses.	Observed standard deviation for difference in ICIQ-FLUTS = 7.
		Observed retention rate of 63 %.
2. What factors influenced eligibility and what proportion of those patients approached were eligible?	37 % of those approached and assessed screened positive.	Of 771 women identified and approached, 284 were (37 %) were deemed eligible.
	Main reasons for ineligibility (see Table 2) were as follows:	
	• Patient has not undergone a course of pelvic floor training	
	• Urge incontinence	
	• Patient did not attend clinic	
3. Was recruitment successful?	Recruitment was slower than anticipated	The mean rate of recruitment per open site month at original sites was 1.9, with a recruitment rate per open site month at the additional full recruiting site of 2.5.
		In 8 months, the PICs did not identify any potentially eligible patients
4. Did eligible participants consent?	Largely achieved, with a recruitment total of 222, representing 93 % of the target of 240.	Of the 284 women who screened positive, 222 (78 %) consented to randomisation
5. Were participants successfully randomised and did randomisation yield equality in groups?	Achieved	Overall, 110 women were randomised to the control arm and 112 to the intervention arm.
		Baseline comparability of the two groups was adequate (Tables 3 and 4)
6. Were blinding procedures adequate?	Not applicable - non-blinded design	
7. Did participants adhere to the intervention?	Largely achieved	In the control group, one woman was found to be ineligible post-randomisation and was withdrawn. The remaining 109 received no IUT.
		In the intervention group, 102 of the 112 women randomised (91 %) received IUT. 2 withdrew because they were unhappy with their allocation, 1 did not attend for cystometry, 3 withdrew for other reasons, and 4 did not receive IUT.
8. Was the intervention acceptable to participants?	Mixed findings; 59 eligible women did not wish to be randomised. Qualitative interviews provided insights into preferences of those consenting to randomisation.	Although most eligible women were willing to be randomised, some had a previously undeclared preference for avoiding IUT and expressed relief at being allocated to the control group. These data will be reported separately elsewhere.
	We did not succeed in interviewing any women who did not consent to the trial.	
9. Was it possible to calculate intervention costs and duration?	We have demonstrated that meaningful and usable data were collected using the instruments we designed for this purpose, and it is feasible to calculate intervention costs based on data collected and from reference costs information. These data will be reported separately elsewhere.	Questionnaires and CRF pages designed to collect relevant information for costs calculation performed reasonably well, with a good response rate and low level of missing data. The average total cost per patient is £1815.26 (SD: 210.39) for ‘IUT’ arm and £1775.37 (SD: 455.38) for ‘no IUT’ arm, based on complete cases only. The mean duration of ‘IUT’ was 40 minutes (SD: 11.028)
10. Were outcome assessments completed?	Mixed picture with poorer completion of outcome assessments at 6 months.	75 % of women returned baseline questionnaires and 63 % returned 6-month questionnaires. Of those who returned questionnaires, some returned incomplete or blank questionnaires
	Bladder diaries and pad use data were poorly completed.	Baseline full completion rates:
		ICIQ-FLUTS: 98 %
		ICIQ-UI SF: 99 %
		ICIQ-LUTSqol: 95 %
		UDI overall score: 84 %
		6-month full completion rates:
		ICIQ-FLUTS: 90 %
		ICIQ-UI SF: 91 %
		ICIQ-LUTSqol: 87 %
		UDI overall score: 81 %
11. Were outcomes measured those that were the most important?		Some evidence from patient interviews that women were less likely to return questionnaires if they were satisfied with the results of treatment
		Lower response rates for instruments towards the end of questionnaire booklet
12. Was retention to the study good?	Rates of loss to follow-up were significant	75 % of women had face-to-face or telephone follow-up after surgical treatment, but only 56 % (63 % of those circulated) returned follow-up questionnaires at 6 months.
13. Were the logistics of running a multicentre trial assessed?	Achieved. Some centres performed better than others, and PICs were not fruitful.	The need to build in adequate time for obtaining global and local approvals was identified
14. Did all components of the protocol work together?	Achieved - components had good synergy	No significant differences identified with trial processes or researchers’ abilities to implement them

CRF, case report form; ICIQ-FLUTS, International consultation on incontinence questionnaire-female lower urinary tracts symptoms; ICIQ-LUTSqol-ICIQ, lower urinary tract symptoms quality of life; ICIQ-UI SF-ICIQ, urinary incontinence-short form; IUT, invasive urodynamic testing; PIC, patient identification centre; UDI, urogenital distress inventory; SD, standard deviation

### Pragmatic multicentre randomised pilot trial

#### Screening, recruitment and randomisation

**Fig. 1:**
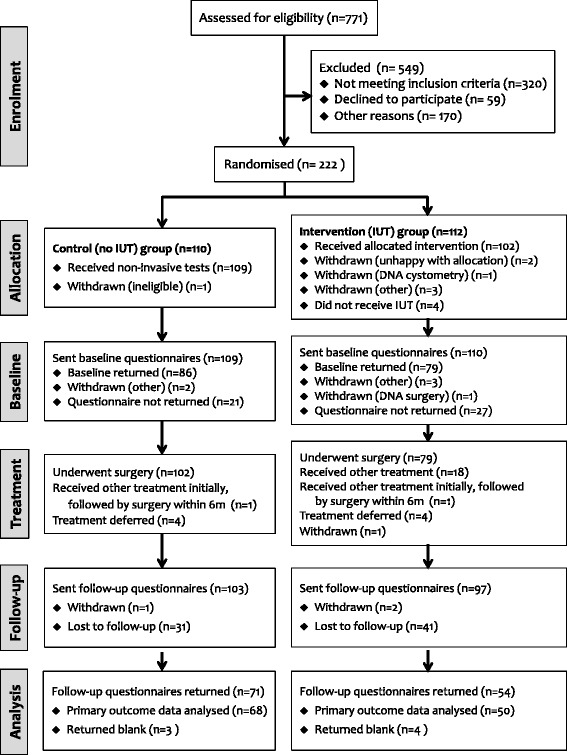
Trial CONSORT flow diagram. IUT, invasive urodynamic testing (intervention) arm; no IUT, no invasive urodynamic testing (control) arm; DNA, did not attend

The screening, recruitment, randomisation and trial follow-up are summarised in the CONSORT diagram shown as Fig. 1. Overall, 771 women were identified and were sent the patient information sheets. Of those, 284 were deemed eligible for the trial, (37 % screen positive). The reasons for non-eligibility, which varied between centres, are shown below in Table 2. One centre accounted for more than half the women screened (399; 52 %).

**Table 2:** Screening & recruitment numbers including screening codes (1-15) for those women not randomised, sorted by overall frequency of reporting of codes

Code	Description	Total	Per cent
11	Patient has not undergone a course of pelvic floor training	105	14 %
14	Urge incontinence	92	12 %
13	Other (give details)	86	11 %
15	Patient did not attend clinic	81	11 %
7	Patient does not wish to participate, include reason if offered	59	8 %
1	Symptomatic utero-vaginal prolapse requiring treatment	40	5 %
8	Clinician feels surgery inappropriate	39	5 %
9	Patient does not wish surgery	21	3 %
2	Previous surgery for urinary incontinence or pelvic organ prolapse	9	1 %
3	Urodynamic investigation within the last three days	7	1 %
10	Patient does not consider her family is complete	6	1 %
4	Neurological disease causing urinary incontinence	1	0 %
5	Current involvement in a conflicting research study	0	0 %
6	Unable to give competent informed consent	0	0 %
12	Study not discussed at clinic visit (please give reason)	3	0 %
	Recruited	222	29 %
	Total screened	771	100 %
	Screened women recruited	222/771	29 %
	Eligible women recruited	222/284	78 %

Of the 284 women screened positive, 222 agreed to randomisation into the trial, giving a trial consent rate of 78 %. This recruitment total (222) represented 93 % of the planned sample size (240) for the pilot trial. Overall, 110 women were randomised to the ‘no IUT’ arm and 112 to the ‘IUT’ arm. Immediately after randomisation, it became apparent that one woman in the ‘no IUT’ arm was ineligible for the trial, and she was withdrawn leaving a total of 221 eligible patients randomised (109 in the ‘no IUT’ arm and 112 in the ‘IUT’ arm).

**Fig. 2:**
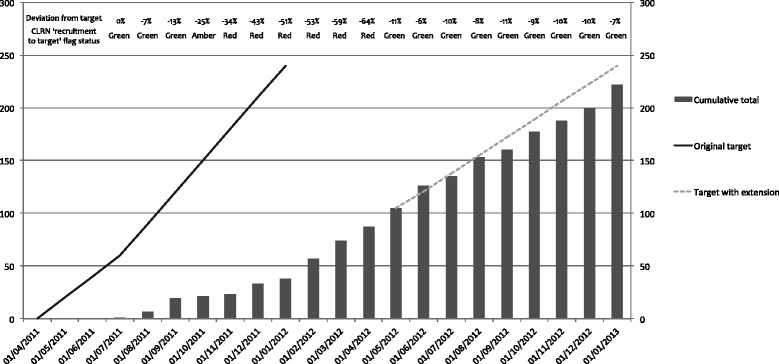
Monthly target and actual recruitment numbers. The original and revised predictions of overall recruitment are shown as continuous and dashed lines, and actual recruitment in histogram; the overall Comprehensive Local Research Network (CLRN) black/red/amber/green flag or ‘recruitment to target’ status is also illustrated

Monthly recruitment is shown in Fig. 2. Regulatory requirements took approximately 3 months longer than anticipated, and recruitment targets were revised accordingly. The rate of accrual over time was significantly less than required; several steps were introduced to improve recruitment, including the incorporation of additional clinicians at two of the existing sites, and the establishment of an additional full recruiting site and two Participant Identification Centre (PIC) sites; a 9-month unfunded extension to the recruitment period was agreed upon with the study funder. Newsletters reporting the progress of the pilot RCT and regular recruitment updates were provided to clinicians in order to maintain their engagement.

The number of participants recruited per recruiting month (that is, between the completion of all site specific regulatory requirements and the end of the study) varied between 0.4 and 3.9 per month at the original sites (mean 1.9); at the additional full recruiting site this figure was 2.5 per month; the PICs did not identify any potentially eligible patients for referral to a recruiting site in the eight months that they were active.

Table 3 provides the demographic data by trial arm; the consistency of these variables between ‘IUT’ and ‘no IUT’ arms confirms the validity of the randomisation process.

**Table 3:** Summary of demographic data at baseline by trial arm

		IUT	no IUT						
		*n*	%			*n*	%		
Ethnicity									
Caucasian	110	99 %			106	97 %			
Black	0	0 %			0	0 %			
Asian	1	1 %			3	3 %			
Other	0	0 %			0	0 %			
		IUT	no IUT						
		*n*	mean (SD)	median (IQR)	range	*n*	mean (SD)	median (IQR)	range
Age		112	47.1 (9.5)	46.5 (40–52)	29–75	110	46.8 (10.0)	46.5 (40–52)	24–77
BMI		106	29.3 (6.5)	28.3 (24.4–33.7)	20–55	102	27.4 (5.0)	26.8 (23.9–30.7)	18–45

BMI, body mass index; SD, standard deviation; IQR, interquartile range; IUT*,* invasive urodynamic testing (intervention) arm; no IUT, no invasive urodynamic testing (control) arm

#### Retention

Two women in the ‘IUT’ group withdrew because they were unhappy with their allocation. Baseline questionnaires were sent to 219 women and returned by 165 (a 75 % response rate overall, 72 % ‘IUT’ arm and 79 % ‘no IUT’ arm). At the 6-month follow-up, questionnaires were returned by 63 % (125/200), (56 % (54/97) ‘IUT’ arm and 69 % (71/103) ‘no IUT’ arm).

#### Completeness of data collection

Not all women fully completed each questionnaire although missing values within individual scales were few. The columns to the right-hand side of Table 4 show the proportion of each questionnaire or subscale that could be calculated from the data provided.

**Table 4:** Summary of numeric outcome measures by trial arm and data collection time-point

	IUT								no IUT								Overall completion rate^a^			
	Baseline					6 months			Baseline				6 months				Baseline		6 months^b^	
Questionnaire (*possible scores*)	*n*	Mean (SD)	Median (IQR)	Range	*n*	Mean (SD)	Median (IQR)	Range	*n*	Mean (SD)	Median (IQR)	Range	*n*	Mean (SD)	Median (IQR)	Range	Partial *n* (%)	Complete *n* (%)	Partial *n* (%)	Complete *n* (%)
ICIQ-FLUTS Overall score (*0–48*)	77	16.9 (5.7)	17 (13–21)	4–37	47	9.2 (7.5)	8 (4–12)	0–38	85	16.4 (6.3)	16 (11–21)	3–34	66	6.9 (5.0)	6 (3–9)	0–26	3 (2)	162 (98)	5 (4)	113 (90)
Subscales:																				
Filling (*0–16*)	78	4.4 (2.3)	4 (3–6)	0–11	48	3.0 (2.3)	3 (1–4)	0–11	85	4.0 (2.6)	3 (2–6)	0–10	66	2.4 (1.8)	2 (1–3)	0–8	2 (1)	163 (99)	3 (3)	114 (91)
Voiding (*0–12*)	79	1.8 (2.0)	1 (0–3)	0–9	49	2.0 (2.0)	2 (0–3)	0–9	86	1.5 (1.7)	1 (0–2)	0–9	68	2.3 (2.1)	2 (0–4)	0–8	0 (0)	165 (100)	1 (1)	117 (94)
Incontinence * (0–20)*	78	10.8 (3.3)	11 (8–13)	2–19	49	4.0 (4.9)	3 (1–5)	0–20	86	10.8 (3.6)	11 (8–13)	2–19	68	2.3 (3.1)	2 (0–3)	0–16	1 (1)	164 (99)	1 (1)	117 (94)
ICIQ-UI SF (*0–21*)	78	14.0 (3.7)	14 (12–16)	4–21	49	5.3 (6.0)	3 (0–8)	0–21	85	14.1 (3.8)	15 (12–17)	4–21	65	3.3 (4.5)	1 (0–4)	0–18	2 (1)	163 (99)	3 (3)	114 (91)
ICIQ-LUTSqol (*19–76*)	73	46.8 (10.9)	47 (40–52)	26–74	44	26.7 (12.3)	22 (20–28)	19–76	84	48.5 (11.7)	46 (39–58)	30–72	65	25.3 (9.6)	21 (20–28)	19–65	8 (5)	157 (95)	9 (7)	109 (87)
UDI																				
Overall score (*0-300*)	64	133.3 (43.5)	133.5 (109–159)	25–245	42	49.1 (44.1)	37.1 (17–69)	0–191	74	130.1 (43.8)	125.8 (96–162)	50–227	59	33.9 (39.7)	24.2 (4–46)	0–150	27 (16)	138 (84)	17 (14)	101 (81)
Subscales:																				
Stress (*0–100*)	76	82.9 (21.0)	87.5 (75–100)	25–100	50	24.5 (26.1)	25 (0–38)	0–100	80	80.2 (21.2)	87.5 (63–100)	38–100	65	18.1 (27.0)	0 (0–25)	0–100	6 (4)	156 (95)	2 (2)	115 (92)
Irritative (*0–100*)	71	38.4 (25.4)	33.3 (17–54)	0–100	48	16.5 (20.5)	8.3 (0–25)	0–100	80	33.7 (24.3)	31.3 (17–50)	0–92	64	10.0 (13.3)	4.2 (0–17)	0–54	13 (8)	151 (91)	6 (5)	112 (90)
Obstructive/ discomfort (*0–100*)	68	17.6 (17.6)	13.6 (6–23)	0–73	43	10.9 (15.1)	4.6 (0–18)	0–64	80	14.8 (14.2)	13.6 (3–20)	0–61	64	8.9 (12.4)	2.3 (0–14)	0–57	17 (10)	148 (90)	11 (9)	107 (86)

ICIQ-FLUTS, International Consultation on Incontinence Female Lower Urinary Tract Symptoms questionnaire; UDI, Urogenital Distress Inventory; *SD* Standard deviation; *IQR* Interquartile range*;* IUT*,* invasive urodynamic testing (intervention) arm; no IUT, no invasive urodynamic testing (control) arm

^a^Complete responses are defined as women who completed all questions on the particular questionnaire scale, and partial responses as those who completed at least one question but did not fully complete the particular scale

^b^In addition to complete and partial responses, there were seven completely blank questionnaires among the 6-month responses

#### Comparison of responders and non-responders to 6-month questionnaire

Given the high rate of non-response to the 6-month questionnaires, a comparison of responders and non-responders was made on the basis of their clinical follow-up. A total of 135 women had a postoperative follow-up visit documented on the study database; 93 actually attended an outpatient clinic, and 42 had a review by telephone (routine practice in three of the centres).

Of the 125 women who returned follow-up questionnaires at 6 months after treatment, 83 had clinical follow-up; of these, 12/83 (14.5 %) described bothersome urinary symptoms, and 9/83 (10.8 %) had clinically significant examination findings. Of the 81 who failed to return follow-up questionnaires at 6 months, 52 had clinical follow-up, of whom 5/52 (9.6 %) described significant urinary symptoms, and 4/52 (7.7 %) had clinically significant examination findings.

Whilst those women returning the 6-month questionnaires had bothersome symptoms or clinically significant examination findings at clinical review somewhat more often than those failing to do so, the numbers do not allow meaningful statistical comparison.

#### Questionnaire data

### Baseline

Table 4 shows the distribution of the questionnaire scales at baseline by trial arm. The distribution of ICIQ-FLUTS total score at baseline was fairly symmetrical with a mean of 16.9 (SD 5.7) in the ‘IUT’ arm and 16.4 (SD 6.3) in the ‘no IUT’ arm. The distributions of the other scales and subscales were similarly well matched between the ‘IUT’ and ‘no IUT’ arms and were fairly symmetrical.

### Six-month follow-up

Table 4 also shows the distribution of the questionnaire scales at the 6-month follow-up by trial arm. For all scales, typical scores were much lower than at baseline. It is difficult to interpret any difference in mean scores between baseline and the 6-month follow-up from Table 4, because of the small sample size and the number of women who provided baseline data but for whom no 6-month questionnaire data are available. Table 5 shows the distribution of the paired changes in scale scores for those women who had completed both questionnaires. It can be seen that the mean change in ICIQ-FLUTS total score was 7.8 in the ‘IUT’ arm and 9.3 in the ‘no IUT’ arm. Typically, there was a marked drop in these scores over 6 months, but little difference in the mean changes between the trial arms; this pattern was also seen in the other four scales, although no formal comparison between arms is appropriate in a pilot study.

**Table 5:** Summary statistics for paired changes in scale scores (from baseline to 6 months)

Questionnaire	*n*	Mean (SD)	Median (IQR)	Range
‘IUT’ arm				
ICIQ-FLUTS - Overall score	31	7.8 (5.9)	7 (4 to 15)	-5 to +18
ICIQ-UI SF	34	8.9 (6.0)	11 (4 to 13)	-3 to +16
ICIQ-LUTSqol	29	20.0 (11.4)	23 (12 to 28)	-5 to +41
UDI - Overall score	27	79.5 (45.5)	75 (51 to 122)	-21 to +161
‘no IUT’ arm				
ICIQ-FLUTS -Overall score	48	9.3 (7.3)	10.5 (5.5 to 15)	-9 to +22
ICIQ-UI SF	49	10.2 (5.8)	11 (6 to 15)	-4 to +21
ICIQ-LUTSqol	47	23.7 (13.9)	23 (14 to 35)	-3 to +50
UDI - Overall score	41	94.1 (55.3)	92 (70 to 117)	-66 to +221

ICIQ-FLUTS, International Consultation on Incontinence modular questionnaire - Female Lower Urinary Tract Symptoms questionnaire; ICIQ-UI SF, ICIQ Urinary Incontinence Short Form questionnaire; ICIQ-LUTSqol, ICIQ Lower Urinary Tract Symptoms quality of life questionnaire; UDI, Urogenital Distress Inventory; SD, standard deviation; IQR, interquartile range; IUT, invasive urodynamic testing (intervention) arm; *no IUT,* no invasive urodynamic testing (control) arm

#### Treatment data

In the ‘IUT’ arm, 82 women (80 %) received surgery, compared to 103 (95 %) in the ‘no IUT’ arm. The distributions of operation type, grade of surgeon, anaesthetic technique and use of antibiotic prophylaxis were similar between the trial arms.

One woman in the ‘no IUT’ arm and four (4 %) in the ‘IUT’ arm decided to defer any treatment initially (designated as ‘w*atchful waiting’*). A further 15 women (15 %) in the ‘IUT’ arm underwent lifestyle changes or other non-surgical treatments. As routine in continence management, more than one lifestyle change was commonly documented, and other non-surgical treatments were often used in combination; 28 treatments were applied in these 15 women. Despite prior (unsuccessful) completion of a course of supervised pelvic floor muscle training (PFMT) being an inclusion criterion for the trial, six women underwent further PFMT alone (*n*=2) or in combination with other non-surgical treatments (*n*=4).

#### Adverse and serious adverse events

Only two serious adverse events were reported. One woman in the ‘IUT’ arm experienced bleeding from the sub-urethral incision 12 days after surgery and one woman in the control arm was treated for breast cancer by mastectomy shortly after her surgery within the trial; whilst the first was clearly related to the incontinence treatment, neither event was categorised as being related to the trial intervention (IUT).

In addition, 23 adverse events were reported in 22 women; these included three operative bladder injuries (3/185=1.6 % perforation rate) and two vaginal injuries. Six episodes of urinary tract infection (UTI) were reported, two in the ‘IUT’ arm and four in the ‘no IUT’ arm; all occurred following surgery, and none occurred immediately after IUT.

#### Calculation of potential sample size of definitive trial

Based upon the trial results, the study team decided that differences of 2, 3 or 4 units on the ICIQ-FLUTS scale would be realistic and potentially clinically important differences that might be achieved.

Given these estimates of effect size, a standard deviation of 7 for paired changes between baseline and follow-up, Type I error of 5 % and Type 2 error of 10 %, total sample size estimates for any definitive trial fall between approximately 200 and 900 women recruited (Table 6). These estimates are considerably less than calculations based upon data in the most recent Cochrane review of urodynamics, which indicate that a sample size of over 1,600 per arm would be required to address this question [24]. With a recruitment rate of 78 %, recruitment of between 200 and 900 would require between approximately 300 and 1200 eligible women to be approached; in turn, with a screen positive rate of 37 %, this would mean between approximately 800 and 3,000 women would need to be identified for screening for eligibility; these ranges depend upon the effect size.

**Table 6:** Total numbers necessary in definitive trial when analysis compares mean changes in ICIQ-FLUTS total score over six months

	Difference to be detected		
	2	3	4
Number of RESPONSES to primary outcome	516	230	130
Number of RECRUITED patients	922	410	232
Number of eligible women APPROACHED	1182	526	298
Number of women SCREENED for eligibility	3194	1422	806

ICIQ-FLUTS, International Consultation on Incontinence modular questionnaires - Female Lower Urinary Tract Symptoms questionnaire

### Patient interview study

All 59 eligible women who declined to participate in the pilot trial were invited to interview but none was willing.

A diverse sample of 111 pilot trial participants was invited to take part in the interview study, including participants from different study sites, the two study arms, a wide range of ages, and those who did and did not complete all follow-up. A total of 36 women indicated they were willing to be interviewed, but of these, two withdrew from the interview study before the interview could be arranged and another had moved and so was no longer covered by our research governance approvals. Of the remaining 33 women, 29 were interviewed before saturation of themes was reached, and the last four were not interviewed as they were from groups already well represented in the sample. Interviewees were between 35 and 75 years of age, came from five of the seven full trial centres, and included participants from both ‘IUT’ (16) and ‘no IUT’ (13) arms.

#### The invitation to participate, and reasons for agreeing

Women’s first reactions to receiving the invitation to participate in the pilot study were almost exclusively positive. The decision to take part was commonly made quickly and easily, and very few reported feeling the need to talk with family or friends as part of the decision-making process.

*WAS IT AN EASY DECISION TO MAKE?*

*Yes, very.*

*DID YOU MAKE IT ON YOUR OWN?*

*Yes, (Participant 10)*

As is commonly found in other studies [61–63], many women’s reasons for participation were altruistic and included wanting to help research, to help others with the same condition, and to make some form of repayment for the help and treatment they were receiving.

Participating in the pilot did not seem to require a lot from them, so no particular participation burden was perceived.

*She explained it very clearly and said all it is basically is just to monitor how many times you go to the toilet, and how much you drink, and roughly how much your output was. And to me I thought that wasn’t a big problem. Only a few minutes of your time in your day, just to keep track. (Participant 04)*

#### The information provided about the study

Reactions to the written information were mostly positive - it was regarded as clear and informative, and there was enough information for women to be able to make a decision about taking part. The short version was sufficient for some and the flow diagram was popular. Others liked to have the fuller detail in the longer version. Overall, most people found it helpful, describing it as easy to read, informative, and pitched at the right level.

*So everything was really well explained you know, so yeah I mean I can’t fault it really, no I was well impressed with it all. (Participant 25)*

The use participants made of the material varied - some read it once only or just skimmed it, others read it more than once, and a small number did additional research about the study on the internet.

*I think I just read it, I didn’t take too much in I think, I think I was just so looking forward to getting my operation that is all I was really erm… really bothered about. I don’t think I read too much about the ins and outs of the study. (Participant 20)*

*Basically I just went on-line and looked at the various things and just erm… just looked at the study. (Participant 15)*

Some were happy with the verbal information at the time of their consultation and paid little attention to the written material, particularly the longer version.

*Personally I wouldn’t bother with the big one, I think that there is enough information, and if you get good medical staff to start with like I did, who actually took the time to go through it with you and say this is what this says, now read it on there, erm… so I think if you get that then you certainly don’t need the bigger one. (Participant 07)*

Suggestions for how the information might be improved were limited but included keeping it as short and concise as possible and distributing it prior to the consultation because some women reported feeling anxious at the consultation and did not initially pay much attention to the information. Given that some women valued the verbal information they received from clinical staff more than the written information, being able to go to the consultation with questions prepared may have been helpful.

#### Understanding of the study

Participants’ understanding of the study was broadly good, although there were some cases in which people appeared confused about the overall aim. Overall, there was a generally good understanding that the study was assessing the value of a particular diagnostic test rather than the treatment they would ultimately receive. Many talked explicitly about how, while participation in the study could influence the route they took to treatment, it was ultimately unlikely to change the final outcome. Establishing this was often important to securing their participation.

*I remember asking him ‘so if I don’t have the test will it have any effect on any treatment I have, and will it have any effect on you deciding what I need?’ No he said, it was purely for this investigation. (Participant 22)*

Not all participants understood the study in this way, though. A small number, when asked to explain what they thought the study was about, did focus on the subsequent treatment rather than the invasive testing.

*I think it's about finding the right appropriate erm… ways forward to treat people with urinary problems. Erm… whether surgery or invasive treatment is appropriate or whether there is another kind of treatment that might be more beneficial. (Participant 17)*

The principle of random allocation to one of two possible groups was generally well understood. There were, however, a small number who thought that participation in the study automatically meant they would avoid the invasive tests.

*DID YOU THINK THERE WAS A POSSIBILITY THAT YOU MIGHT HAVE THE INVASIVE TESTS?*

*Erm…no I think the registrar said to me if I signed up for the study I wouldn’t have them. (Participant 08)*

#### Experiences of study participation

The first set of questionnaires participants were asked to complete at baseline was generally described as simple to fill in, easy to understand, and straightforward.

*HOW DID YOU FIND THE QUESTIONNAIRES YOU WERE ASKED TO COMPLETE AT THE BEGINNING?*

*Simple.*

*WERE THEY TIME CONSUMING AT ALL?*

*No not particularly. (Participant 01)*

A few minor issues were raised: there wasn’t always a box to tick that was applicable to them, some questions were hard to answer (for example, when asked to work out costs or where judgement was called for), and some thought the questions were a little repetitive.

*Sometimes there wasn't, you know how there were tick boxes kind of thing, it…none of those were really the answer that I wanted to give. (Participant 11)*

There were also some comments on the practical challenges associated with measuring urine output for the bladder diary.

*I found it more difficult to collect the urine. You know to get down to it and have clear, clear days to get on with it. (Participant 18)*

The second set of questionnaires sent out 6 months after treatment were similarly felt to be relatively simple to complete. However, given that many had had successful treatment and now had few, if any, symptoms to report, the questions did not always seem relevant. Indeed, one participant reported having called the study office to check she had been sent the right questionnaires, and others were a little concerned it might appear that they had not completed the questionnaires at all because so much was not now applicable to them.

*I actually sent it back with absolutely nothing on it at all because it said ‘have you been to visit the doctor in 6 months’, and I hadn't and it said go to the next section, and go to the next section and so by the end of it, there was nothing on it and I sent it back completely blank and I thought they will think I have not bothered filling this in. (Participant 14)*

While some actually found completing the 6-month questionnaires quite enjoyable (as it underlined for them how successful the treatment had been), others reported finding them burdensome and irrelevant now they had few or no symptoms to report.

*Not relevant at all, not to me anyway. Yes, because I mean the problem was solved then so, why harp on about how many pads am I wearing now because I don't wear them, simple as that, nothing. (Participant 09)*

This seemed particularly to apply to the bladder diaries.

*It did want another bladder diary I think afterwards and I have not completed the bladder diary because I just didn't get round to it to be honest with you. I had it in my bag to take to work with me and I just didn't get round to doing it. (Participant 21)*

## Discussion

The findings and implications of this pilot are considered in subsequent sections across a number of aspects of the trial design [60]. In terms of the ADePT approach, the problems identified related to aspects of trial process and were therefore classified as Type A - issues likely to be a problem only for a trial, but not in the real world [45].

Overall, the logistics and study procedures were seen to be adequate and functional in most areas, and important insights were gained to inform the design and efficient conduct of any future definitive trial. These include the following: allowing a realistic time frame for regulatory approval and site start-up, clarity over inclusion/exclusions, modifying screening processes, reduction in secondary outcomes, modification to patient questionnaire booklets and bladder diaries, and employing a range of strategies to retain trial centre engagement (for example, website, newsletters, recruitment updates).

### Eligibility, recruitment, consent and randomisation

We found that 37 % of the women screened were deemed eligible for the trial. This figure varied between centres, as did the declared reasons for ineligibility. More than half of all the women screened were from one centre. It is likely that the assiduousness of recruiters and interpretation of eligibility criteria differed between centres. Running screening training exercises might be considered for a future definitive trial to ensure similar screening standards and practices and an ‘assumed eligibility’ approach in all centres. This should be feasible, for example, by ‘clustering’ centres geographically and carrying out training exercises alongside site setup visits; we do not, however, have evidence of the effectiveness of this proposed solution.

Recruitment was initially slow and was more successful in some centres than others. Recruitment was initially delayed by the fact that ethical and regulatory requirements for a multi-centre study took longer than expected, and any definitive trial should determine and allow a realistic timeframe for this.

Once approvals were in place, it was necessary to expand the number of planned centres and clinicians within centres to meet recruitment targets; this highlights the need for rigorous and realistic site feasibility assessments prior to site selection and setting and on-going monitoring of individual site targets.

Whilst there is little high-quality evidence to support their use [64], a range of strategies was used to retain trial centre engagement such as regular recruitment updates and newsletters. However we were eventually able to recruit patients from all our study centres in sufficient numbers to confirm that recruitment was feasible.

Of those women who screened positive, 78 % consented to enter the trial. Data from the patient interviews suggested that most women reacted positively to the invitation to take part, and found the information provided about the study to be clear. There was no clear preference for either the shorter or longer version of the patient information sheet. The principle of random allocation to one of two trial arms was generally well understood by participants. The randomisation procedure led to similar sized groups that were well balanced on baseline variables.

### Compliance with and acceptability of intervention

Most patients received the ‘IUT’ (91 %) or ‘no IUT’ group tests (99 %) to which they were allocated. However, two patients withdrew from the trial because they were unhappy to be randomised to the ‘IUT’ arm, one failed to attend the appointment for IUT, and four other patients in the IUT arm did not undergo invasive tests for unspecified reasons.

### Outcome assessment, selection of most appropriate outcomes and participant retention

Completion rates were relatively high for all questionnaires, and they had a similar rate and spread of missing items. Rates of loss to follow-up after treatment were significant, however, and whilst 75 % of women had either face-to-face or telephone follow-up (typically at two to three months) after surgical treatment, only 56 % (63 % of those circulated) returned follow-up questionnaires at 6 months.

It is recognised that the completion of questionnaires can be burdensome for participants [65], and this may be particularly the case for those with few or no symptoms. We found some evidence in the patient interview study to suggest that women were less likely to return questionnaires if they were satisfied with the results of their treatment, which may account for the number of blank questionnaires returned at six months.

In any future definitive trial it would be necessary to ensure a higher questionnaire response rate. The UDI was the fourth instrument in a booklet of six questionnaires in total, and had a slightly lower completion rate at both baseline and 6 months. The questions in ICIQ-UI SF overlap considerably with those in the longer ICIQ-FLUTS and so we recommend omitting both UDI and ICIQ-UI SF from any definitive trial to reduce respondent burden. We anticipate that this may improve completion of the remaining items. Greater emphasis needs to be placed on the importance of returning a completed questionnaire even in the absence of any remaining symptoms. Alternative modes of completion for follow-up questionnaires (for example, telephone or web based) and providing incentives to return questionnaires are further evidence-based strategies that might enhance retention rates for data collection [66, 67].

Bladder diary data and pad test use were poorly completed in our pilot. This may be because many of the women would have completed similar diaries or frequency/volume charts earlier in their continence assessment, because it may be seen as rather more intrusive than simple questionnaire responses, or because it is possible that the diary design resulted in inconsistent completion of pad-use data. The trial recruitment process enrolled only women with SUI or stress-predominant MUI, the diary data did not show any evidence of abnormal urinary frequency or nocturia, and there appeared to be no change at 6 months in either arm (other than in pad-use). In order to increase the completion rate of incontinence episode data, diary data and pad use might be omitted or modified in any definitive trial.

Alternative modes of completion for follow-up questionnaires, such as by telephone or online, and the provision of modest incentives to return the questionnaires [66, 67] are further evidence-based strategies that might enhance retention rates for data collection. A further possibility is to link questionnaire completion at follow-up to the face-to-face clinic review, thereby allowing a check by a research nurse or trial coordinator of item completion before patients leave the clinic area; however, this would have required a change to the current practice of some units, and risked the pragmatic nature of the trial.

### Sample size calculation for a definitive trial

Sample size estimates were calculated for target differences of 2, 3, and 4 units in ICIQ-FLUTS, using the standard deviation of the primary outcome data from the pilot trial. However, a monograph on ways of specifying a target difference for a trial recommended that estimates of sample size should be determined by more than one approach [68]. In any definitive trial, the following data sources might be amongst those considered:

1. Clinician opinion
2. Data from the external pilot trial
3. A value of information study (not included here, but forming part of a separate report) [42].

A survey update in June 2013 of consultant members of BSUG and BAUS-SFNUU sought their views on what constitutes a minimum clinically significant target difference in ICIQ-FLUTS combined score. However, the ICIQ-FLUTS scale has not been used in many published studies to date, and, perhaps because it is therefore not familiar, only 50 % of consultants responding expressed an opinion. There was no evidence of a common opinion: given a choice of seven ranges of the scale to define a clinically important difference (from 1–4 to >24), all these ranges were chosen by at least one clinician, with the modal range being 9 to 12. In separate discussions, members of the study team did not find it easy to choose a target difference based on the limited use of the scale so far. The current lack of data from published trials using ICIQ-FLUTS, and therefore evidence on which to base expert judgement, casts some doubt of the usefulness of a survey of experts in this situation.

When the pilot trial results became available, it was apparent that the distribution of the ICIQ-FLUTS total score at 6 months and the difference between the scores at baseline and 6 months typically had low values. The mean score (SD) at 6 months in the ‘no-IUT’ arm was 6.9 (5.0) and the mean change between baseline and 6 months was 9.3 (7.3). It was apparent, therefore, that it is not realistic to expect differences in mean outcomes between trial arms in the order of 9 to 12 units, as proposed in clinician survey responses. Based upon the trial results, the study team decided that differences of 2, 3 or 4 units would be realistic differences that might be achieved in any comparison of an intervention for women eligible for a future trial.

Given the observed standard deviations, these target differences of 2, 3 or 4 units are equivalent to standardised effect sizes of 0.29, 0.43 and 0.57 when comparing mean changes in score over 6 months. In contrast, a difference of 9 to 12 units would equate to a standardised effect size of 1.5 to 2, which is a very large difference; many trials are planned on a standardised effect size of around 0.5. Cohen has suggested that standardised differences of 0.2, 0.5 and 0.8 correspond to ‘small’, ’medium’ and ‘large’ effect sizes [20].

If a study is planned on the basis of a ‘realistic’ value for the target difference, then consideration has to be made of whether this is also a ‘clinically important’ difference. If it is clear that this is not a ‘clinically important’ difference, then there are real doubts as to whether the trial should take place. It was felt that a difference of around three units would also be of clinical interest since a decrease of this level would equate to complete recovery for one of the symptoms assessed in the ICIQ-FLUTS score.

In this pilot trial, we identified 771 women for screening from seven centres over the course of 114 centre screening months (approximately 6.8 women/centre/screening month). Extrapolation of these figures would require 120 to 480 centre screening months to achieve the recruitment of 200 to 900 women. This would mean four to 20 centres recruiting for approximately 30 months or six to 30 centres recruiting over 18 months.

## Conclusions

Overall, the pilot trial can be considered a success, and a definitive trial is feasible and remains necessary. The study procedures were seen to be adequate and functional in most areas, and important insights were gained to inform the design and efficient conduct of a future definitive trial.

Lessons were learned in how to manage the time needed to bring multiple centres online through the UK regulatory process; likely variation in recruitment from the multiple centres has been observed and the importance of standardised and assiduous screening recognised; and effective methods of communication to keep staff engaged through the lifetime of a long study have been rehearsed and refined. Refinements in the data collection process that will improve the quantity and quality of the data for a definitive trial have been identified.

Although recruitment was initially slow, patients were recruited from all study centres in sufficient numbers to confirm that recruitment is feasible and that women are happy to engage with the study objectives and be randomised. Participants were very positive about the study, and in particular allayed fears over whether research to ‘test a test’ would be seen as important. The interviews also offered suggestions as to how the experience of participation could be improved and data collection maximised.

Based upon a range of target differences derived from the observed clinical outcomes in this pilot RCT, any definitive trial may need to recruit between 200 and 900 women. With recruitment rates also based upon the pilot RCT, this would mean four to 20 centres recruiting for approximately 30 months or six to 30 centres recruiting over 18 months.

## Abbreviations

BAUS-SFNUU: British Association of Urological Surgeons, Section of Female, Neurological, and Urodynamic Urology
BSUG: British Society of Urogynaecology
CLRN: Comprehensive Local Research Network
CRF: case report form
DO: Detrusor overactivity
HES: Hospital Episode Statistics
ICER: incremental cost-effectiveness ratio
ICIQ-FLUTS: International Consultation on Incontinence Modular Questionnaires: Female Lower Urinary Tract Symptoms questionnaire
ICIQ-LUTSqol: ICIQ Lower Urinary Tract Symptoms Quality of Life Questionnaire
ICIQ-UI SF: ICIQ Urinary Incontinence Short Form Questionnaire
IUT: Invasive Urodynamic Testing
MUI: mixed urinary incontinence
NIHR: National Institute for Health Research
OAB: overactive bladder
PCQ: participant costs questionnaire
PFMT: pelvic floor muscle training
PIL: Patient Information Leaflet
QALY: quality of life year
RCT: randomised controlled trial
SUI: stress urinary incontinence
UDI: Urogenital Distress Inventory
UI: urinary incontinence
